# Shedding of GPP130 by PC7 and Furin: Potential Implication in Lung Cancer Progression

**DOI:** 10.3390/ijms26136164

**Published:** 2025-06-26

**Authors:** Priyanka Prabhala, Stephanie Duval, Alexandra Evagelidis, Maïlys Le Dévéhat, Vatsal Sachan, Nabil G. Seidah

**Affiliations:** Laboratory of Biochemical Neuroendocrinology, Montreal Clinical Research Institute (IRCM), affiliated to the University of Montreal, Montreal, QC H2W 1R7, Canada; pprabhala@oicr.on.ca (P.P.); stephanie.duval.4@ulaval.ca (S.D.); alexandra.evagelidis@ircm.qc.ca (A.E.); mailys.ledevehat@ircm.qc.ca (M.L.D.); vatsal.sachan@ircm.qc.ca (V.S.)

**Keywords:** GPP130, GOLIM4 proprotein convertases, PC7, furin, post-translational modifications, proteolysis, Cys-palmitoylation, lung cancer

## Abstract

From a previously performed proteomics screen, GPP130, or Golgi phosphoprotein of 130 kDa, was identified as a potential substrate of the proprotein convertase 7 (PC7; PCSK7). GPP130 is a type-II transmembrane protein with a luminal domain containing endosomal and Golgi-retrieval determinants, enabling a unique trafficking route. Most of the previous work on GPP130 relates to its binding and retrograde trafficking of the Shiga toxin. However, its cellular biology and its biochemical characterization remain understudied. Recently, GPP130 was reported to be implicated in cell cycle progression and cell proliferation in head and neck cancer cells. This led us to analyze the cBioPortal for Cancer Genomics, revealing that the GPP130/GOLIM4 gene is amplified in many cancers, including lung, ovarian, and cervical. This observation led us to use the A549 lung cancer cell line to investigate the growth-regulating roles of endogenous and overexpressed GPP130 and to analyze the impact of its cleavage/shedding by PC7 and/or Furin on cellular growth. Our cell-based assays suggest that GPP130 is a novel pro-protein convertase substrate that increases cell proliferation in A549, SKOV3, and HeLa cells, and that the latter activity is enhanced following its cleavage by PC7 and/or Furin into a membrane-bound N-terminal product and secreted C-terminal fragments. This novel work sheds light on the cell biology of the poorly characterized GPP130, its proliferative activity, and modulation upon its shedding by PC7 and Furin in lung cancer progression.

## 1. Introduction

The proprotein convertases are a mammalian family of nine secretory serine proteases related to bacterial subtilases and yeast kexin (PCs or PCSKs), implicated in various biological processes as well as in pathologies such as cancer/metastasis [1]. Proprotein convertases (PCs) mediate the cleavage and activation of a diverse range of precursor proteins [1]. The first seven members of this family specifically recognize and cleave (↓) substrates at single or paired basic amino acid residues within the consensus motif (R/K)-(2X)n-(R/K)↓, where *n* = 0, 1, 2, or 3 spacer aa, and X is a variable amino acids (aa) [1]. Cellular and animal studies demonstrated the implication of Furin [2,3], PC7 [4], PACE4 [5,6,7], and PCSK9 [8,9] in cancer/metastasis. So far, in the clinic, therapies targeting the last member of the family, PCSK9, are highly successful in the treatment of hypercholesterolemia and beyond [10,11]. The physiological roles of the ubiquitously expressed PC7 (gene PCSK7), the seventh member of the family [12], remained obscure for a long time—however, very recently, Sachan et al. [13] demonstrated that in mice, liver PC7 plays a critical role in enhancing apolipoprotein B (apoB) levels, and the silencing of its expression provided a promising treatment for diet-induced metabolic dysfunction-associated steatotic liver disease (MASLD), a devastating pathology that affects at least 25% the population worldwide [14].

**Figure 1:**
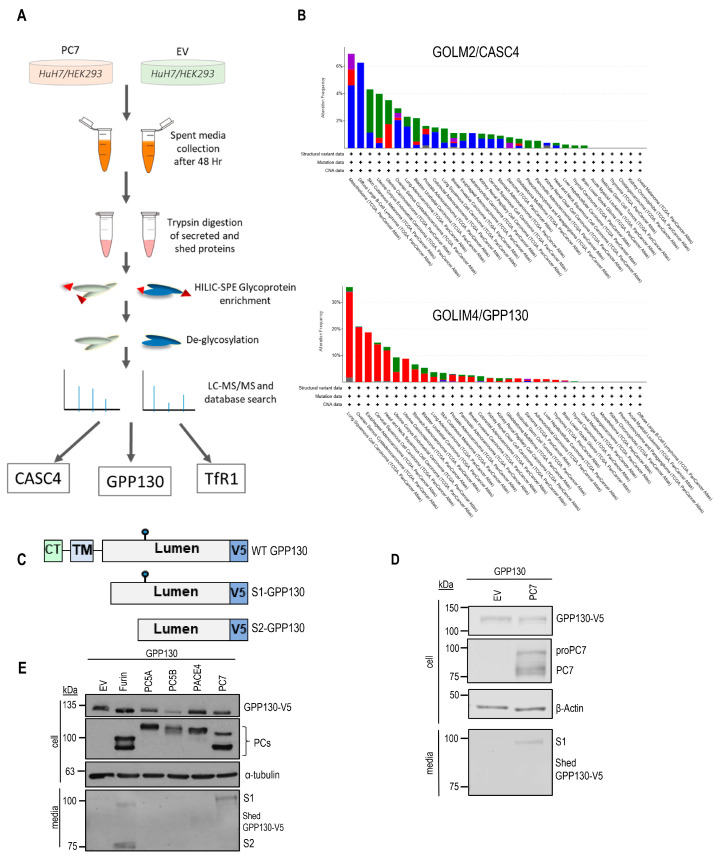
PC7 and Furin cleave GPP130. (**A**) A schematic explaining the identification of three PC7 substrates using mass spectrometry analysis. (**B**) Analysis of genetic alterations in the GOLM2 gene (protein product: CASC4) and GOLIM4 (protein product: GPP130) using cBioPortal data. The figure was plotted using the cBioPortal website and depicts the frequency and type of genetic alterations for the two genes. (**C**) Schematic representation of human Golgi Phosphoprotein of 130 kDa (GPP130) protein. Depicted are the cytosolic tail (CT), the transmembrane domain (TM), the luminal domain, and the C-terminal V5-tag. The blue circle depicts a potential N-glycosylation site. (**D**) Western blot analysis of cell lysates and media from HEK293 cells expressing GPP130-V5 with either pIRES-empty vector (EV) or human PC7 [*n* = 3]. (**E**) Western blot analysis of cell lysates and media from HEK293 cells expressing GPP130-V5 with all the basic aa PCs or pIRES-empty vectors [*n* = 3]. The images shown are representative of three independent experiments.

To identify novel PC7 substrates, we performed a quantitative proteomics screen targeting N-glycosylated polypeptides secreted by hepatic HuH7 cells overexpressing PC7. This analysis revealed two type-II transmembrane proteins—cancer susceptibility candidate 4 (CASC4) and Golgi phosphoprotein 130 (GPP130)—that are shed into soluble forms by PC7 and/or Furin (Figure 1A) [4]. The latter study revealed that CASC4, especially its PC7/Furin-cleaved N-terminal membrane-bound fragment, enhanced the migration and invasion of triple-negative MDA-MB-231 breast cancer cells [4]. Our analysis from the cBioPortal for Cancer Genomics database has shown that the expression of the genes coding for CASC4 and GPP130 are differentially modified in various cancers (Figure 1B). However, GPP130 and its shedding were not functionally characterized, especially since GPP130 expression is amplified in up to 35% of patients with lung cancer and >10% in head-and-neck cancer patients (Figure 1B). Herein, we have filled the knowledge gap on GPP130 and additionally compared the properties of GPP130 to those of CASC4.

GPP130 is a type-II transmembrane Golgi protein comprising a short cytosolic tail, a 20-aa transmembrane domain, and a luminal domain that contains determinants guiding its trafficking itinerary [15,16]. Hence, GPP130 is able to traffic from the cis-Golgi to the plasma membrane and back via the TGN38/46 bypass pathway [17,18]. Further investigations demonstrated that GPP130 traffics to early endosomes where it binds the Shiga toxin internalized into cell surface endosomes, and the complex is then sorted back to the Golgi and retrograde transported to the endoplasmic reticulum (ER) to exert its cytotoxic effects [19,20]. Additionally, manganese (Mn^2+^) treatment has been shown to induce GPP130 oligomerization, thereby redirecting it into multivesicular bodies (MVBs) en route to lysosomal degradation [21,22]. Thus, GPP130 maintains intracellular Mn^2+^ homeostasis by binding excess Mn^2+^ in the Golgi lumen, thereby initiating the routing of Mn^2+^-bound GPP130 to lysosomes for degradation. Indeed, Mn^2+^ treatment inhibits the progression of multiple types of 3q-amplified malignancies by degrading GPP130, resulting in a secretory blockade that interrupts pro-survival autocrine loops and attenuates pro-metastatic processes in the tumor microenvironment [23]. GPP130 is thus considered a Golgi-resident protein with a distinctive trafficking route, facilitating cargo transport within the secretory pathway. Moreover, the GPP130/GOLIM4 gene has been implicated in head and neck cancer through a study that identified it as a downstream target of stromal interaction molecule 1 (STIM1), a Ca^2+^ channel protein involved in store-operated calcium entry (SOCE)—a pathway activated in response to decreased Ca^2+^ levels in the ER [24]. In this study, GPP130 expression was found to be elevated in head and neck cancer cells and was proposed as a downstream target of STIM1 [25]. Furthermore, GPP130 has been implicated in regulating cell cycle progression and proliferation by modulating the expression of MDM2 and CDK6 in these cancer cells [25]. Recently, it was also shown that miR-105-3p acts as an oncogene to promote the proliferation and metastasis of breast cancer cells by silencing GOLIM4 [26] and that targeting miR-942-5p/GPP130 axis suppressed breast cancer malignant behavior [27]. Finally, a non-biased proteomic analysis identified GPP130 as a novel target for the treatment of endometrial cancer [28]. Thus, so far, the available data suggest that depending on the cancer type, GPP130 can act as a tumor suppressor or an oncogene. However, the specific role of GPP130 or its fragments in lung cancer has not been studied.

Because of the amplified expression of GPP130 in lung cancer patients (Figure 1B), in the present study, we concentrated on the ex vivo growth function activity of GPP130 in the lung carcinoma A549 cell line. The functional implications of PC7/Furin in the cleavage of GPP130, as well as its cytosolic tail Cys-palmitoylation, were studied in more detail. We also compared the growth-promoting activities of GPP130 to those of CASC4 and their shed products. The data showed that GPP130 cleavage by PC7 and/or Furin results in enhanced cellular growth, likely due to the production of a secreted soluble form and an N-terminal membrane-bound fragment. The potential Cys-palmitoylation of the latter was found to reduce its growth-promoting activity. Conversely, blocking the PC7/Furin processing of GPP130 results in a substantial reduction in cellular growth. Thus, the inhibition of PC7/Furin-induced shedding of GPP130 may represent a promising strategy for the reduction in lung cancer tumor growth.

## 2. Results

### 2.1. GPP130 Is Cleaved and Shed by PC7 and Furin

Using a mass spectrometry approach consisting of selective enrichment of N-glycosylated secreted polypeptides, performed previously to define novel PC7 substrates [4]. Our group recently identified a type-II transmembrane protein, GPP130, as a potential PC7 candidate substrate (Figure 1A). In the current study, we investigated the functional relevance of GPP130 shedding by PC7. To do so, we first validated that GPP130 is processed by PC7 in HEK293 cells co-expressing cDNAs encoding GPP130 with a V5-tag at its C-terminus (Figure 1C), PC7, or an empty vector control plasmid (Figure 1D). Western blot (WB) analyses revealed that GPP130 is cleaved and shed by PC7 since we observed the release of a ~100 kDa fragment (S1) in the media in the presence of PC7 when using an antibody targeting the V5 tag on its luminal domain, but not in control cells expressing a cDNA encoding an empty vector (EV) (Figure 1D). In addition, co-expression in HEK293 cells of cDNAs encoding GPP130 with each of the basic aa-specific PCs (Furin, PC5A, PC5B, PACE4, and PC7), or an EV control, revealed that only PC7 and Furin cleave and shed GPP130 into the media (Figure 1E). Interestingly, Furin cleaved GPP130 at two sites within its luminal domain, releasing distinct ~100 kDa (S1) and ~75 kDa (S2) fragments into the media (Figure 1E).

**Figure 2:**
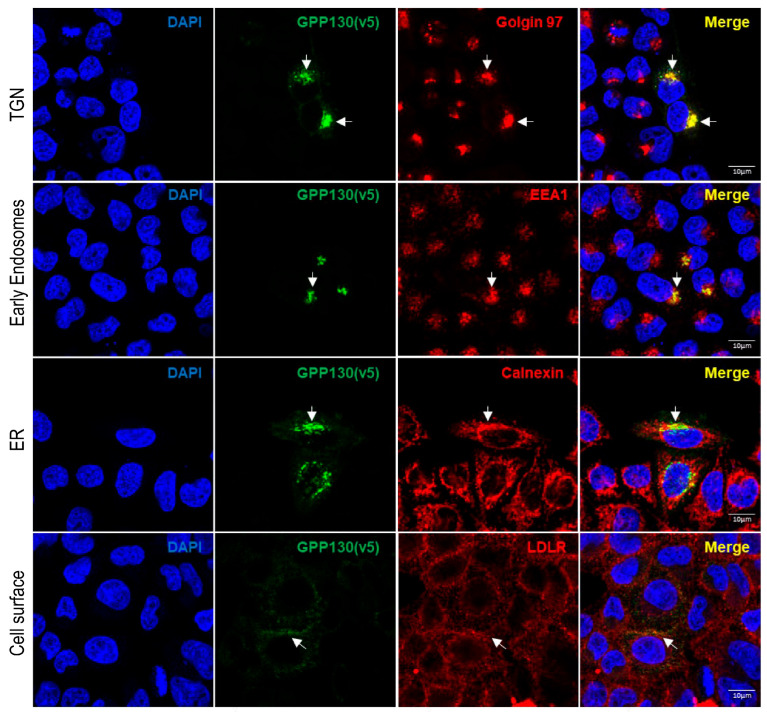
GPP130 is localized primarily in the TGN when overexpressed in HeLa cells. Immunofluorescence analysis of permeabilized HeLa cells overexpressing human GPP130-V5 colocalizing (white arrows) with TGN marker (Golgin-97), early endosome marker (EEA1), endoplasmic reticulum marker (calnexin) and plasma membrane marker low-density lipoprotein receptor (LDLR) (non-permeabilized condition) [*n* = 3]. The images shown are representative of three independent experiments. Scale: 10 µm.

To better understand the trafficking of GPP130, immunofluorescence analysis demonstrated that when overexpressed in HeLa cells, GPP130 colocalizes mainly with the TGN marker Golgin-97, early endosome antigen 1 marker (EEA1), the ER marker calnexin and to some extent with the plasma membrane marker low-density lipoprotein receptor (LDLR) (Figure 2). These results are coherent with previous publications demonstrating that PC7 and Furin activities mainly occur in the TGN, cell surface, and/or endosomal-like structures [29,30].

### 2.2. Identification of GPP130 Sites Shed by PC7 and Furin

**Figure 3:**
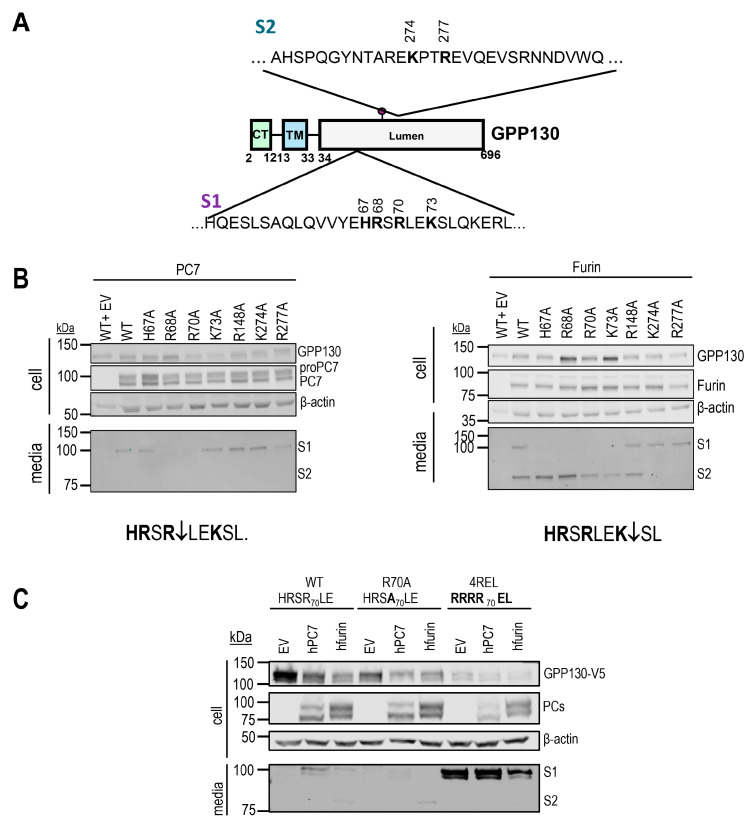
GPP130 cleavage by PC7 and Furin occurs at HRSR70↓LE and HRSRLEK73↓, respectively, and solely at KPTR277↓EV for Furin. (**A**) Schematic representation of GPP130 cleavage motifs and released fragments. (**B**) Western blot analysis of cell lysates and media from HEK293 cells overexpressing GPP130-V5 WT and different point mutations (H67A, R68A, R70A, K73A, R148A, K274A, or R277A), with human PC7, human Furin, or pIRES-empty vector (EV) [*n* = 3]. (**C**) Western blot analysis and quantifications of cell lysates and media from HEK293 cells overexpressing GPP130-V5 WT, GPP130 4REL (an optimally cleaved mutant), or GPP130 R70A (a PC non-cleavable mutant) co-expressed with human PC7, human Furin, or pIRES-empty vector [*n* = 3]. The images shown are representative of three independent experiments.

To identify GPP130 cleavage sites, we replaced arginines or lysines with alanines within potential consensus cleavage motifs (R/K)-2Xn-(R/K)↓ using site-directed mutagenesis and assessed the shedding of GPP130 by analyzing its release into the media [1]. Western blot analysis of HEK293 cells overexpressing cDNAs encoding wild type (WT) GPP130, its mutant forms (H67A, R68A, R70A, K73A, R148A, K274A or R277A) or empty vector control, demonstrated that the R68A and R70A mutants are resistant to PC7 shedding and that basic residues variants in the stretch from H67A to K73A are resistant to Furin shedding within the motif H67RSRLEK73 (S1-site) (Figure 3A,B). Also, while R148A did not affect PC7 or Furin shedding, K274A and R277A variants of GPP130 were resistant to Furin’s second shedding (S2-site) (Figure 3A,B). These results suggest that GPP130 is cleaved (S1 site) at HRSR_70_↓ by PC7 and HRSR_70_LEK_73_↓ by Furin but that only Furin cleaves GPP130 (S2 site) at KPTR_277_↓EV (Figure 3B). To further emphasize that these are the cleavage sites, we generated mutant proteins harboring an optimized PC-recognition site [1] RRRR_71_EL (hereafter called 4REL).

WB analysis demonstrated that this motif is indeed the cleavage site because the expression in HEK293 cells of cDNAs coding for the GPP130-4REL mutant generated a soluble form of the protein secreted into the media, even when co-expressed with an EV control, with the same molecular size as the WT construct co-expressed with PC7 or Furin (Figure 3C). Furthermore, the expression of the R70A mutant prevented cleavage at the S1-site by both PC7 and Furin but still allowed the Furin S2-site cleavage (Figure 3B,C). These data suggest that the S2 cleavage by Furin does not depend on the prior generation of the S1 fragment and that both S1 and S2 fragment formation by Furin could occur independently.

### 2.3. GPP130 Is Cleaved in Post-ER Acidic Compartments

**Figure 4:**
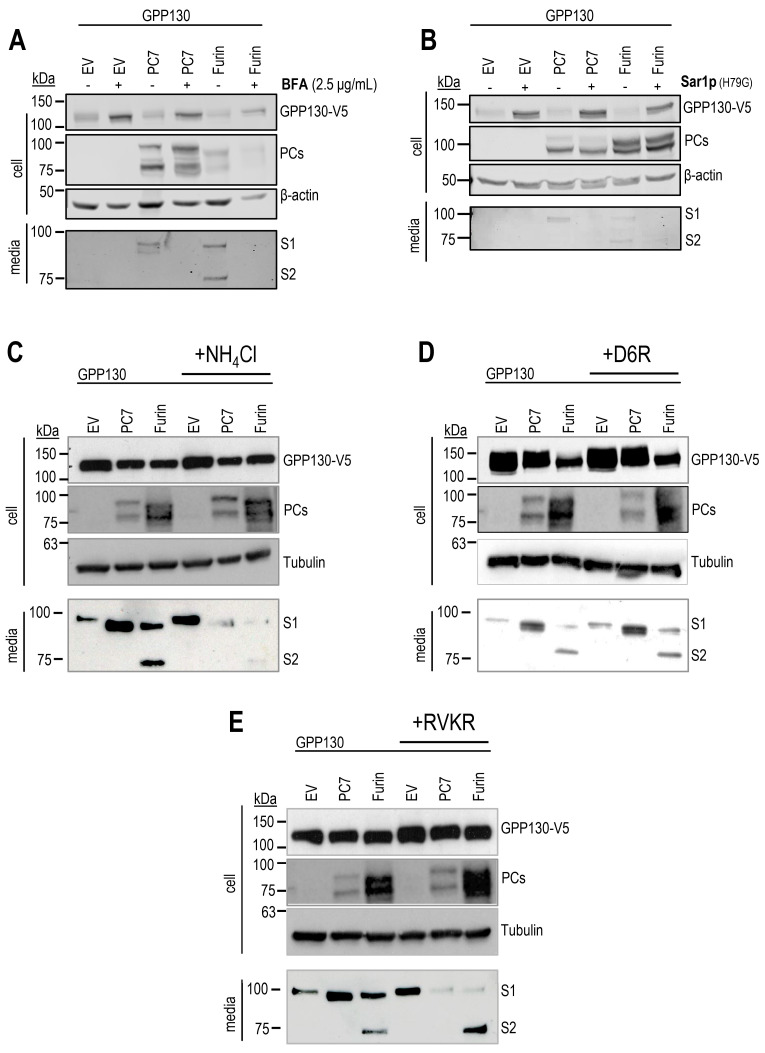
GPP130 is cleaved in a post-ER acidic compartment by PC7 and Furin. (**A**,**B**) Western blot analysis of cell lysates and media from HEK293 cells overexpressing GPP130-V5 WT and human PC7, human Furin, or with pIRES-empty vector (vector) in the presence or absence of BFA (**A**) or (**B**) Sar1p(H79G) variant [*n* = 3]. Notice the secretion of the s1-fragment by PC7 and Furin and the S2 fragment by Furin. (**C**–**E**) Western blot analysis and quantifications of cell lysates and media from HEK293 cells overexpressing GPP130-V5 WT, and human PC7, human Furin or with pIRES-empty vector (vector) in the presence or absence of NH_4_Cl (**C**), D6R (**D**) or dec-RVKR-cmk (RVKR) (**E**) [*n* = 3]. The images shown are representative of three independent experiments.

We took advantage of different inhibitors and mutant proteins to determine which cellular compartments GPP130 is cleaved by PC7 and Furin. In HEK293 cells, WB analysis showed that the shedding of GPP130 by PC7 and Furin was abrogated when treated with Brefeldin A (BFA) (Figure 4A), an inhibitor that blocks the transport of proteins from the ER to the cis-Golgi [31]. Shedding was also inhibited when we overexpressed PC7 or Furin in combination with a dominant negative Sar1p (H79G) mutant protein (Figure 4B), a GTP-restricted mutant that blocks COPII vesicle formation [32]. We conclude that the processing of GPP130 occurs in post-ER compartments. It is interesting to note that GPP130 intracellular levels are increased upon both BFA treatment and the co-expression of the Sar1p (H79G) mutant (Figure 4A,B). This suggests that these conditions are, in part, blocking the ‘normal’ degradative route taken by GPP130 when overexpressed in cells. We next used the alkalizing agent ammonium chloride (NH_4_Cl), which blocks the acidification of intracellular compartments, to test if the shedding occurs in acidic pH compartments, as previously described [30]. Accordingly, treatment of HEK293 cells with NH_4_Cl prevented GPP130 shedding at both S1 and S2 by PC7 and Furin (Figure 4C), suggesting that cleavage at both sites occurs in the acidic environment of the TGN and/or endosomes.

We next compared the effects of the cell surface (non-cell-permeable) inhibitor hexapeptide D-Arg6 (D6R) and the cell-permeable pan-PC general inhibitor decanoyl-RVKR-cmk (RVKR) [33]. The data demonstrated that D6R did not affect either of the two cleavages, supporting the NH_4_Cl data that suggested that neither cleavages occur at the neutral pH of the cell surface (Figure 4D). In contrast, treatment with the irreversible inhibitor dec-RVKR-cmk, which alkalinizes the active site His* in Furin and PC7, abolished almost completely S1 shedding by both PC7 and Furin (Figure 4E), emphasizing that the S1-processing is PC-specific. Unexpectedly, the S2-cleavage by overexpressed Furin is not affected by dec-RVKR-cmk, suggesting that it may occur in an acidic environment not readily accessible to dec-RVKR-cmk, possibly late endosomes and/or multivesicular bodies, where GPP130 is known to traffic [21]. Indeed, Furin activity is enriched >3-fold in endosomes compared to the TGN, and yet it is 10-fold less inhibited by dec-RVKR-cmk [29]. The reduced inhibition of Furin by dec-RVKR-cmk in endosomes may also be related to the higher acidity of late endosomes whereupon the Furin active site His* would be more protonated and hence resistant to irreversible alkylation by dec-RVKR-cmk. Taken together, these data suggest that GPP130 is cleaved at S1 by both Furin and PC7 in post-ER acidic compartments, e.g., TGN and/or endosomes, and at S2 by Furin in a distinct acidic compartment.

### 2.4. GPP130 Influences Cell Proliferation in Lung Cancer-, Ovarian Cancer- and Cervical Cancer-Derived Cell Lines

**Figure 5:**
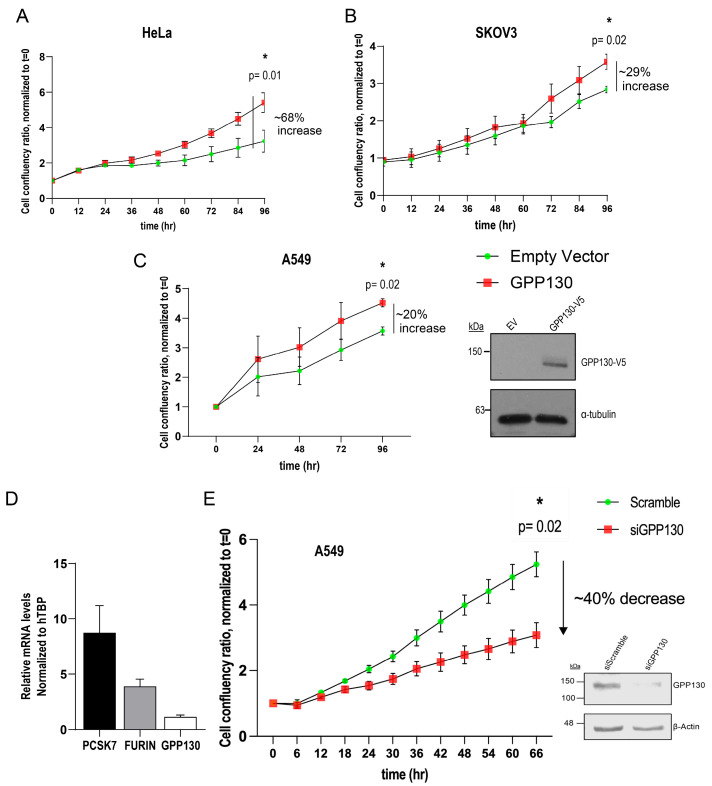
GPP130 enhances the proliferation of HeLa, SKOV3, and A549 cells. (**A**–**C**) Incucyte analysis of the cellular proliferation of human HeLa (**A**), SKOV3 (**B**), and A549 (**C**) cells overexpressing GPP130. The data are normalized to day 0, and proliferation is followed by 96 h [*n* = 3]. (**D**) qPCR analysis of the mRNA levels of PCSK7, Furin, and GPP130 in naive A549 cells, normalized to the housekeeping gene human TATA-binding protein (hTBP) [*n* = 3]. (**E**) Incucyte analysis of the cellular proliferation of A549 cells lacking GPP130 (siGPP130) [*n* = 3]. The efficiency of the siRNA is shown in (**E**), and the overexpressed levels of GPP130 in A549 cells are shown in (**C**) [*n* = 3]. The statistically significant results (*p* < 0.05) at the 96 h timepoint are depicted. The Western blot images shown are representative of three independent experiments. Asterisk mean it is statistically significant *p* = 0.01 or 0.02.

The GOLIM4/GPP130 gene enhances cell proliferation via the modulation of key regulators of the cell cycle in head-and-neck cancer cells [25]. We therefore wanted to investigate whether GPP130 shedding, as seen in the current study, could also influence cell proliferation. Hence, we first interrogated a genomics database (cBioPortal for Cancer Genomics) and noted that the GOLIM4/GPP130 gene is amplified in up to ~35% of lung cancer patients, ~20% of ovarian cancer patients, ~15% of cervical cancer and in >10% of head-and-neck cancer patients (Figure 1B). Accordingly, we selected lung cancer-derived A549, ovarian cancer-derived SKOV3, and cervical cancer-derived HeLa cells to perform cell proliferation assays. Although the overexpression of GPP130 cDNA in these cell lines resulted in an increase in cell proliferation (Figure 5A–C), we chose to continue with A549 cells owing to the high alteration frequency of GPP130 in lung cancers (Figure 1B) and the fact that these cells endogenously express PC7, Furin, and GPP130 (Figure 5D). Our results showed that upon treatment of A549 cells with siRNAs to GPP130 that effectively reduced its protein expression by >80%, the cells exhibited a significant decrease in cellular proliferation (~40%; Figure 5E). In contrast, upon overexpression of GPP130 cDNA, we observed a significant increase in cell proliferation (~20%; Figure 5C).

### 2.5. Identification of Critical Domains and Palmitoylation of GPP130 Implicated in Enhanced Proliferation

**Figure 6:**
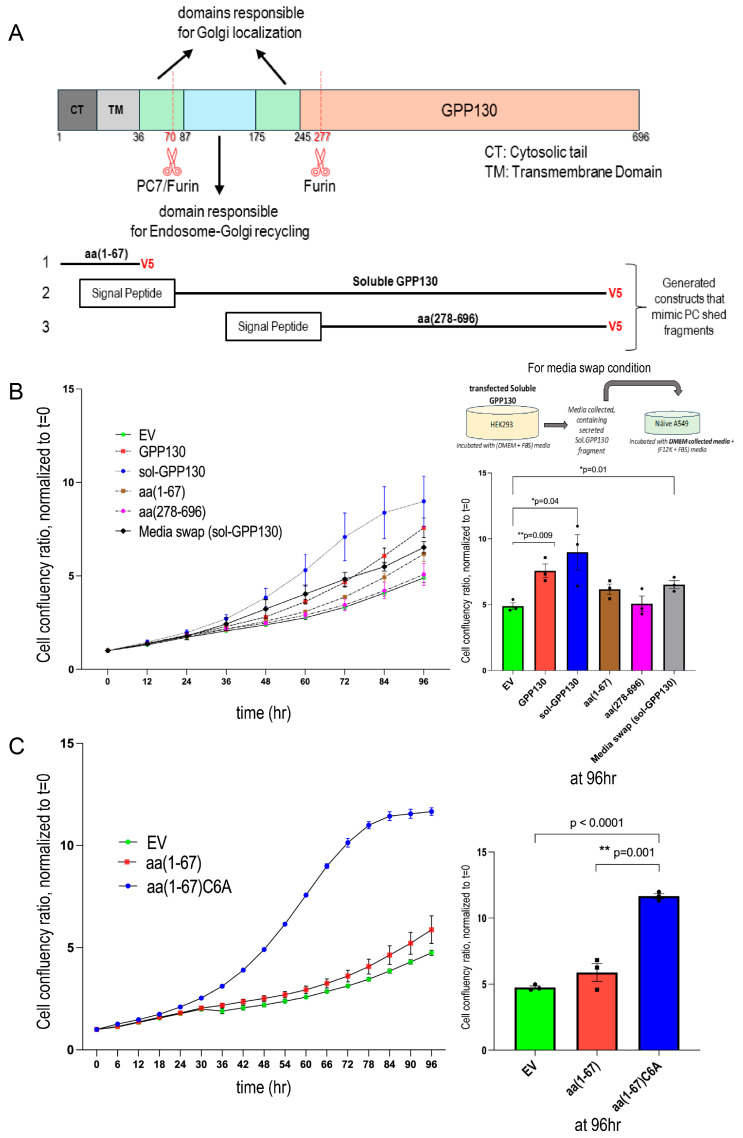
(**A**,**B**) GPP130 domains implicated in enhancing the proliferation of A549 cells and the paracrine effect of GPP130 shedding. (**A**) Schematic diagram of the various constructs used. (**B**) Incucyte analysis of cellular proliferation of A549 cells overexpressing GPP130-FL, soluble GPP130, the N-terminal fragment (aa 1-67) generated by S1-cleavage and the C-terminal product of the S2-cleavage (aa 278-696) or with pIRES-empty vector (EV) [*n* = 3]. Additionally, cellular proliferation of naïve A549 cells incubated with the conditioned media of HEK293 over expressing sol-GPP130 compared to A549 cells overexpressing soluble GPP130 or the pIRES-empty vector (EV) is also depicted (experimental design shown in the inlet). (**C**) Palmitoylation of GPP130 regulates the proliferation of A549 cells. Incucyte analysis of cellular proliferation of naïve A549 cells overexpressing soluble GPP130 aa(1-67) compared to its non-palmitoylated C6A mutant or the pIRES-empty vector (EV) [*n* = 3]. The data are normalized to day 0 and proliferation is followed for 96 h. The data are normalized to day 0 and proliferation is followed for 96 h. The statistically significant results (* *p* < 0.05, ** *p* < 0.005) at the 96 h timepoint are depicted as separate bar graphs.

We next wanted to assess if GPP130 shedding by PCs could affect cell proliferation and identify the critical domains in GPP130 that may enhance the proliferation of A549 cells. Thus, according to data obtained in Figure 3, we engineered three constructs to mimic the products generated upon cleavage at the S1 and S2 sites. This included the N-terminal membrane-bound fragment consisting of amino acids aa 1-67 generated upon S1 cleavage and the corresponding aa 71-696 (soluble sol-GPP130), and the soluble secreted Furin-generated S2 product aa 278-696 (Figure 6A).

Compared to the EV control, WT GPP130 and sol-GPP130 enhance the growth of A549 cells by ~35% and ~85%, respectively (Figure 6B). In contrast, aa 1-67 and aa 278-696 did not significantly affect growth. This suggests that the proliferation-promoting domain of GPP130 may reside in aa 71-696.

We next tested whether the secreted sol-GPP130 would have a paracrine growth-enhancing effect. Thus, we either overexpressed a cDNA encoding sol-GPP130 or treated naïve A549 cells with media obtained from HEK293 cells overexpressing sol-GPP130 (media swap, Figure 6B). Even though the extent of enhanced cellular growth by media-swapped sol-GPP130 is smaller than that caused by overexpression of this fragment, clearly, sol-GPP130 in the media could enhance cellular growth in a paracrine fashion (Figure 6B). The lesser effect on cellular growth observed could be due to the different media used for maintaining and producing the conditioned media from HEK293 cells (DMEM media) and that used to maintain A549 cells (F12K media), leading us to dilute in a ratio of 1:3 the media of HEK293 cells (and hence the secreted sol-GPP130) by the F12K media.

Furthermore, it is possible that expression of sol-GPP130 (artificially) could result in an intracellular activity on growth that is distinct from that seen extracellularly upon the addition of sol-GPP130 to the cells. Indeed, only in the overexpressed sol-GPP130 condition can we detect this form in the cells (Supplementary Figure S1), but not in overexpressed full-length GPP130 + PC7 or Furin (Figure 1 and Figure 4). This suggests a lack of accumulation in cells of the shed form produced by PC7 or Furin cleavage, which may be rapidly secreted. The extracellular form of sol-GPP130 may interact with a receptor(s) at the cell surface and affect growth accordingly, whereas the intracellular GPP130 may have a different mechanism to control cellular growth.

We also noticed a Cys6 in the cytosolic tail of GPP130, a potential site of S-palmitoylation, wherein a possible covalent attachment of palmitic acid could take place. Accordingly, we generated a C6A mutant of GPP130 aa(1-67), namely, GPP130 aa(1-67)C6A that could not be palmitoylated. Notably, the A549 cellular proliferation data show that upon blocking the potential Cys-palmitoylation of the N-terminal membrane-bound domain, there is a very significant ~2.4-fold increase in proliferation (Figure 6C).

### 2.6. Comparative Growth of A549 Cells Overexpressing GPP130 or CASC4 Constructs

**Figure 7:**
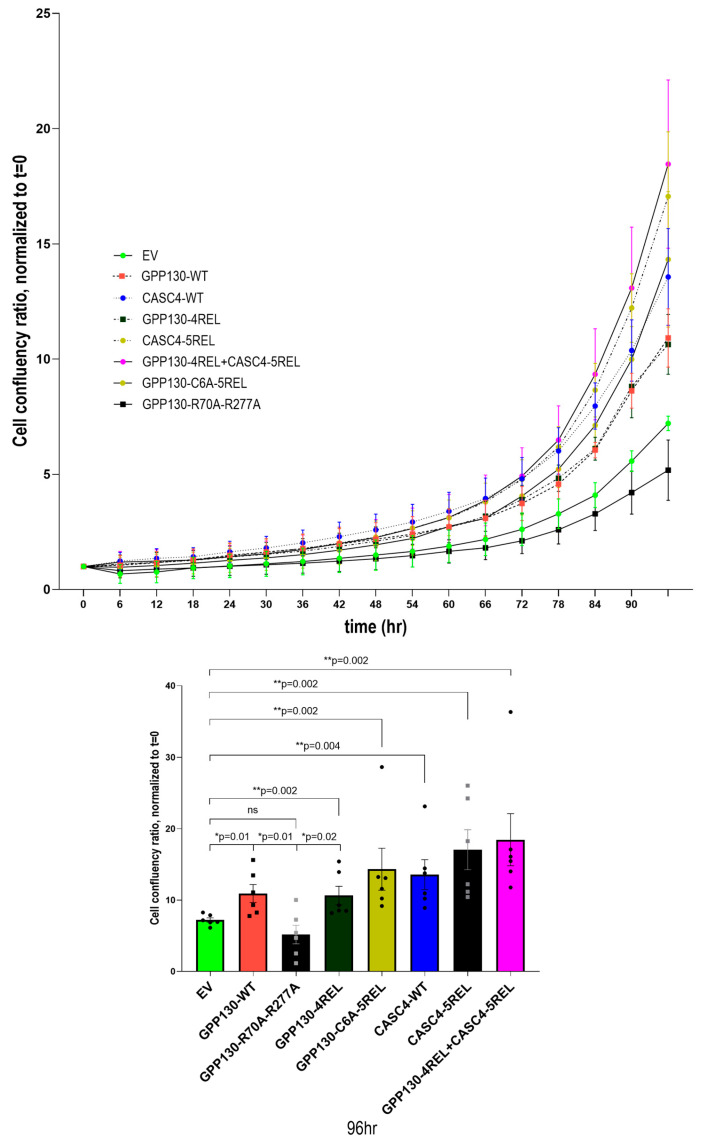
Shedding of GPP130 and CASC4 enhances the proliferation of A549 cells. Incucyte analysis of cellular proliferation of A549 cells overexpressing pIRES-empty vector (EV) or WT GPP130, GPP130-R70A-R277A, GPP130-4REL, GPP130-C6A-5REL, WT CASC4, CASC4-5REL, GPP130-4REL + CASC4-5REL. The data are normalized to day 0 and proliferation is followed for 96 h. The statistically significant results (* *p* < 0.05, ** *p* < 0.005) at the 96 h timepoint are depicted as separate bar graphs [*n* = 3], ns means not significant.

As observed in all our previous experiments, overexpression of WT GPP130 enhanced proliferation by an average of ~40% (Figure 7). In contrast, compared to WT GPP130, the absence of shedding at both S1 and S2 (GPP130 R70A + R270A) resulted in a proliferation like that obtained with an empty vector (Figure 7), suggesting that complete abrogation of shedding prevents the proliferation induced by GPP130. Notably, the GPP-4REL mutant did not significantly enhance proliferation above that observed with WT GPP130 (Figure 7). This is likely because in A549 cells, overexpression of GPP130-4REL results in a very effective S1 cleavage, which is subsequently cleaved with the predominant formation of the non-proliferative S2 fragment aa 278-696 (Figure 6B), Indeed, Western blot analysis reveals the presence of only the S2 fragment when GPP-4REL is overexpressed in A549 cells (Supplementary Figure S1). This contrasts with the overexpression of GPP130-4REL in HEK293 cells that leads to the predominant generation of the S1 fragment without significant cleavage at S2 (Figure 3C). In our previous study, we observed that efficient shedding of CASC4 (variant CASC4-5REL) enhances triple-negative breast cancer MDA-MB-231 cells migration and invasion [4]. However, the proliferative effect of this variant was not studied. In the present study, we compared the proliferative activity of CASC4 overexpression and its efficient shedding (CASC4-5REL) in lung cancer A549 cells. The data show that CASC4 significantly enhances the proliferation of A549 cells compared to EV (Figure 7). We also observed a trend revealing that CASC4-induced proliferation is ~24% higher compared to that resulting from GPP130 overexpression (Figure 7). However, CASC4-5REL only slightly enhances proliferation above that observed with WT CASC4, as opposed to a more robust increase in migration/invasion previously observed [4]. It should be noted that CASC4 is only cleaved once at Arg_66_↓ [4], whereas GPP130 exhibits two shedding sites (S1 and S2). Finally, the combination of GPP130-4REL and CASC4-5REL led to a proliferation phenotype like that of CASC4-5REL alone, supporting the above data on the lack of added pro-proliferative activity of GPP130-4REL compared to WT GPP130.

## 3. Discussion

GPP130 is a Golgi resident protein with a unique trafficking pathway. Most of the literature covering GPP130 is related to its role in transporting the Shiga toxin intracellularly [22]. In addition, investigations aiming at targeting GPP130 trafficking for therapeutic uses in the context of Shiga toxin infections have demonstrated that this protein is sensitive to Mn^2+^ and can be rerouted for degradation in lysosomes following Mn^2+^ treatment [21]. However, other biological roles of GPP130, including its cell biology, remain unknown. Notably, a group interested in Ca^2+^ signaling during tumor growth has recently highlighted the importance of GPP130 in cell cycle progression and showed that the knockdown of GPP130 severely impacts cell proliferation in head-and-neck cancer cells [25].

Herein, we demonstrated that GPP130 is shed by PC7 and Furin at two distinct sites (S1 and S2) in its luminal domain, which would be expected to have dramatic consequences on the efficient binding and transport of the Shiga toxin. Site-directed mutagenesis revealed that the S1 site cleaved by PC7, and Furin occurs at Arg_70_↓ and Lys_73_↓, respectively (Figure 3B), and that Furin is the only convertase that cleaves at Arg_277_↓ (S2 site). Notably, site-directed mutagenesis revealed that the PC7 cleavage at S1 occurs at H**R**S**R**_70_↓ and that Arg_70_ at P1 and Arg_68_ at P3 are critical but not His_67_ at P4 (Figure 3B). It is unusual that a P3 Arg would be critical for PC-cleavage, but His at P4 is present in other PC-substrates such as mouse pro-epidermal growth factor (proEGF) [34].

We also demonstrated that the independent cleavages at S1 and S2 occur in distinct intracellular acidic compartments. Cleavage at S1 is inhibitable by dec-RVKR-cmk and NH_4_Cl, suggesting that both PC7 and Furin process GPP130 in the TGN and/or acidic endosomes, as expected from the localization of PC7 activity in endosomes [30] and of Furin activity in the TGN and endosomes [35]. In contrast, the S2 cleavage by Furin seems to occur in dec-RVKR-cmk resistant but NH_4_Cl-sensitive acidic compartment(s) (Figure 4). Interestingly, in the absence of overexpressed PC7 or Furin, GPP130 seems to be shed at a site closer to the transmembrane domain, possibly by an endogenous cell surface metalloprotease (more active in the presence of NH_4_Cl), as suggested by the upward shift in S1 on Western blots (compare EV versus PC7 or Furin in Figure 4C–E).

Finally, one drawback of this study is the need to overexpress PC7 or Furin to detect GPP130 shedding by endogenous proteases in either HEK293 or A549 cells. The identification of a lung cancer cell line with high enough endogenous PC7, Furin, and GPP130 levels may support our conclusions. Indeed, analysis of the Cancer Genome Atlas database revealed that in certain tumors, PC7, Furin and GPP130/GOLIM4 are highly expressed (Supplementary Figure S2).

(https://www.proteinatlas.org/ENSG00000160613-PCSK7/cancer;
https://www.proteinatlas.org/ENSG00000140564-FURIN;
https://www.proteinatlas.org/ENSG00000173905-GOLIM4/cancer, accessed on 21 May 2025).

It is possible that endogenous GPP130 shedding may be readily observed in cells derived from such cancers that overexpress Furin and/or PC7 endogenously, as observed in our previous report in breast cancer tumors, e.g., MDA-MB-231 [4], which is what this study is trying to mimic using PC7 or Furin overexpression in model cell lines.

We next tested the effect of silencing endogenous GPP130 (siRNA) or its overexpression on the cellular proliferation of A549 cells (Figure 5). As shown for head and neck cancer, it is plausible that the knockdown of GPP130 also leads to the perturbation of Stromal Interaction Molecule 1 (STIM1)-GOLIM4 signal axis in A549 cells, thereby reducing the cellular growth [25]. Our data extends this GPP130-induced pro-proliferative activity to lung cancer A549 cells and reveals that it is the GPP130 shedding at S1 by PC7 and/or Furin that enhances such proliferative activity (Figure 6). In contrast, the GPP130 R70A-R277A variant that is completely resistant to shedding showed no proliferative activity at all (Figure 7).

Structure-function studies demonstrated that two domains in GPP130 regulate its pro-proliferative activity: (1) The N-terminal domain aa(1-67), in which the prevention of the likely reversible Cys_6_-palmitoylation aa(1-67)C6A [36] of GPP130 [37] significantly enhances cellular growth (Figure 6C); and (2) the N-terminal domain of the secreted shed GPP130 at S1 [aa(71-696)] (Figure 6B) that encompasses domains responsible for endosome to Golgi recycling and TGN localization (Figure 6A). Since this fragment seems to enhance cellular proliferation upon its incubation with naïve A549 cells (Figure 6B), future studies may identify a putative cell-surface binding partner/receptor and its subsequent signaling pathway. Interestingly, sortilin has been shown to interact with the luminal domain of GPP130 [38], it is plausible that shed GPP130 may bind sortilin at the cell surface and that triggers a cascade of events resulting in higher cellular proliferation [39].

By comparison, CASC4 was also shown to be shed at a single site by Furin and PC7, and the generated N-terminal transmembrane fragment (aa 1-66) also enhances cellular migration and invasion [4]. Thus, both GPP130 and CASC4 have N-terminal membrane-bound domains that enhance cellular proliferation and/or migration, which may be implicated in cancer progression. Indeed, the overexpression of CASC4 or the efficiently shed CASC4-5REL variant in A549 cells led to increased proliferation compared to EV (Figure 7). The presence of multiple basic residues in the cytosolic tails of both GPP130 and CASC4 suggests that they may act as nuclear localization signals that could sort them to the nucleus [40] if released into the cytosol following a putative g-secretase cleavage within their transmembrane domain, as observed with various transmembrane proteins [41]. Previous studies have shown that the dysregulation of palmitoylation is indeed critically involved in many cancers [42]. This dysregulation, combined with GPP130 shedding via PC7/Furin, may explain the proliferative effects of GPP130.

In conclusion, we showed for the first time that GPP130 is cleaved by PC7 and Furin and that this shedding could lead to the release of an ectodomain fragment, which may enhance cell proliferation in a similar fashion to that of another family member protein, GP73 that is highly oncogenic [43,44]. The present study sheds light on a poorly characterized Golgi-resident substrate of PC7 and Furin, and future work will help to elucidate its biological roles, binding partners, and how PCs modulate its functions. Our findings highlight the functional significance of the shedding of GPP130 by PC7 and Furin and its effect on the proliferation of human lung carcinoma A549 cells. Our data suggest that PC7 and/or Furin, which are implicated in many cancers via their enzymatic activity [3,4,45], may also enhance the shedding of novel cancer growth factors such as GPP130 and CASC4. Future studies should validate the functional importance and consequences of their silencing/inhibition in the treatment of aggressive cancers and their associated metastasis.

## 4. Materials and Methods

### 4.1. Plasmids

Human GPP130 WT, GPP130 aa(1-67), GPP130 aa(278-696), soluble GPP130 (sol-GPP130, Signal peptide—aa(71-696)-lacking the transmembrane domain; ΔTM), GPP130 SP-aa(71-277), full-length human PC7, full-length human Furin cDNAs were cloned V5 tag at the C-terminus, into pIRES-EGFP vector (Clontech, Mountain View, CA, USA). Site-directed mutagenesis was used to generate G2A, C6A, G2AC6A, R70A, GPP130 (RSR70LEK) variant RRRR71EL (called 4REL) to generate a Furin/PC7 optimized cleavage site, and a PC uncleavable mutant, GPP130 (R70AR277A).

### 4.2. Cell Culture, Transfections, and Cell Treatments

A549 cells were grown in Kaighn’s Modification of Ham’s F-12 Medium (F12K, Invitrogen, Burlington, ON. Canada) supplemented with 10% Fetal Bovine Serum (Wisent Inc, Saint-Jean-Baptiste, QC, Canada) and 1% Penicillin-Streptomycin (MilliporeSigma, Oakville, ON, Canada). HEK293 and HeLa cells were grown in Dulbecco’s modified Eagle’s medium (DMEM, Invitrogen) with 10% FBS, Invitrogen. SKOV3 cells were grown in McCoy’s 5A medium with 10% FBS. All cells were maintained at 37 °C under 5% CO_2_. HEK293 cells were co-transfected with equimolar quantities of each plasmid using Jetprime Polyplus (Sartorius Canada Inc, Oakville, ON, Canada), whereas A549 cells were transfected with equimolar quantities of each plasmid using FuGene HD, using the manufacturer’s instructions.

### 4.3. Cell Treatments

At 24 h post-transfection, cells were washed in serum-free medium followed by an additional 24 h with serum-free media (SFM) alone or SFM with 20 μM NH_4_Cl or 10 μM D6R, 75 μM dec-RVKR-cm. Sol-GPP130 conditioned media were produced following HEK293 cell transfection with 2 µg cDNA of sol-GPP130 or empty vector constructs using FuGene HD as described above in complete DMEM media. The media were collected 48 h post-transfection, centrifuged for 5 min (12,000 rpm at 4 °C) and analyzed for sol-GPP130 presence using Western blotting. The media were then stored at −80 °C until use. For media swap, cells were washed with PBS once, followed by the addition of 250 µL conditioned media and 750 µL complete F12K media after which the cells were subjected to proliferation analysis.

### 4.4. Immunofluorescence

Cells were grown on coverslips and fixed with warm paraformaldehyde (4%) for 10 min and permeabilized with PBS 1X + Triton 0.1%. Following permeabilization or PBS incubation, cells were stained for primary antibodies: anti-EEA1 (Abcam Inc., Toronto, ON, Canada), anti-Golgin 97 (Santa Cruz Biotechnology, Mississauga, ON, Canada), anti-LDLR (R&D Systems, Toronto, ON, Canada), anti-calnexin (Abcam Inc.), or anti-V5 (Invitrogen Canada Inc.) for 1 h followed by incubation with fluorescent corresponding secondary antibodies for 1 h in the dark. Coverslips were then mounted on a microscope slide (Fisher Scientific, Ottawa, ON, Canada) with ProLong Gold antifade with DAPI (Invitrogen) to stain the nucleus. For cell surface labeling, the permeabilization step was skipped, keeping the rest of the protocol as mentioned above.

### 4.5. Western Blot Analysis and Antibodies Used

Proteins were extracted in RIPA buffer with cocktails of protease inhibitors (Roche Canada, Laval, QC, Canada). Bradford assay was used to evaluate protein concentrations. Approximately 50 µg protein was loaded for each sample and the levels of the housekeeping gene α-tubulin or β-actin were used as loading controls. Proteins were resolved on SDS-PAGE and blotted onto nitrocellulose membranes. The following primary and secondary antibodies were used to incubate the membrane: Mouse anti-V5 (1:5000, Invitrogen Canada Inc.), rabbit anti-β-actin (1:5000, MilliporeSigma), rabbit anti-α-tubulin (1:10,000, Proteintech group, Inc., Rosemont, Il, USA) or horseradish peroxidase (HRP)-conjugated mAb V5 (1:10,000, MilliporeSigma), anti-PC7 (1:1000; cell signaling technology) and anti-Furin (1:5000 ThermoFisher Scientific, St Laurent, QC, Canada). Quantifications were performed using the ChemiDoc imaging system (Biorad, Hercules, CA, USA).

### 4.6. Cell Proliferation Analysis

For cell proliferation assays, cells were seeded into 24 well plates (Greiner, MilliporeSigma) at low cellular density (15,000 cells/well) and placed into the Incucyte imager for up to 96 h. The images were taken by the machine at intervals of 12 h to generate cellular density (% of confluence), and results were graphed over time. Each experiment was performed at least 3 times, and subsequent statistical analysis was performed, wherein ns means *p* > 0.05, * *p* ≤ 0.05, ** *p* ≤ 0.01 and *** *p* ≤ 0.001. Normality and equality of variance of the data were checked using the Shapiro–Wilk test (https://www.statskingdom.com/shapiro-wilk-test-calculator.html, accessed on 21 May 2025) and F-test (https://www.statskingdom.com/220VarF2.html, accessed on 21 May 2025), respectively. The differences between groups were tested using parametric unpaired Student’s *t*-test with Welch’s correction for unequal variance (https://www.graphpad.com/guides/prism/latest/statistics/stat_the_unequal_variance_welch_t_t.htm, accessed on 21 May 2025) or nonparametric Mann–Whitney U test (https://www.socscistatistics.com/tests/mannwhitney/default2.aspx, accessed on 21 May 2025) if required.

For cell proliferation assays, cells were seeded into 24 well plates (Greiner, MilliporeSigma) at low cellular density (15,000 cells/well) and placed into the Incucyte imager for up to 96 h. The images were taken by the machine at intervals of 12 h to generate cellular density (% of confluence), and results were graphed over time. Each experiment was performed at least 3 times, and subsequent statistical analysis was performed, wherein ns means *p* > 0.05, * *p* ≤ 0.05, ** *p* ≤ 0.01 and *** *p* ≤ 0.001. Normality and equality of variance of the data were checked using the Shapiro–Wilk test (https://www.statskingdom.com/shapiro-wilk-test-calculator.html, accessed on 21 May 2025) and F-test (https://www.statskingdom.com/220VarF2.html, accessed on 21 May 2025), respectively. The differences between groups were tested using parametric unpaired Student’s *t*-test with Welch’s correction for unequal variance (https://www.graphpad.com/guides/prism/latest/statistics/stat_the_unequal_variance_welch_t_t.htm, accessed on 21 May 2025) or nonparametric Mann–Whitney U test (https://www.socscistatistics.com/tests/mannwhitney/default2.aspx, accessed on 21 May 2025) if required.

## Data Availability

Data that supports the findings in this study are available from the corresponding authors upon reasonable request.

## Supplementary Materials

The following supporting information can be downloaded at https://www.mdpi.com/article/10.3390/ijms26136164/s1.
