# Supplementary material for: Shedding of GPP130 by PC7 and Furin: Potential Implication in Lung Cancer Progression
Source: Int J Mol Sci. 2025 Jun 26;26(13):6164. doi: 10.3390/ijms26136164 (PMC12250082; doi:10.3390/ijms26136164)
Supplement: Supplementary file 1 [file ijms-26-06164-s001.zip › ijms-3685487-supplementary.pdf]

Supplementary Figures

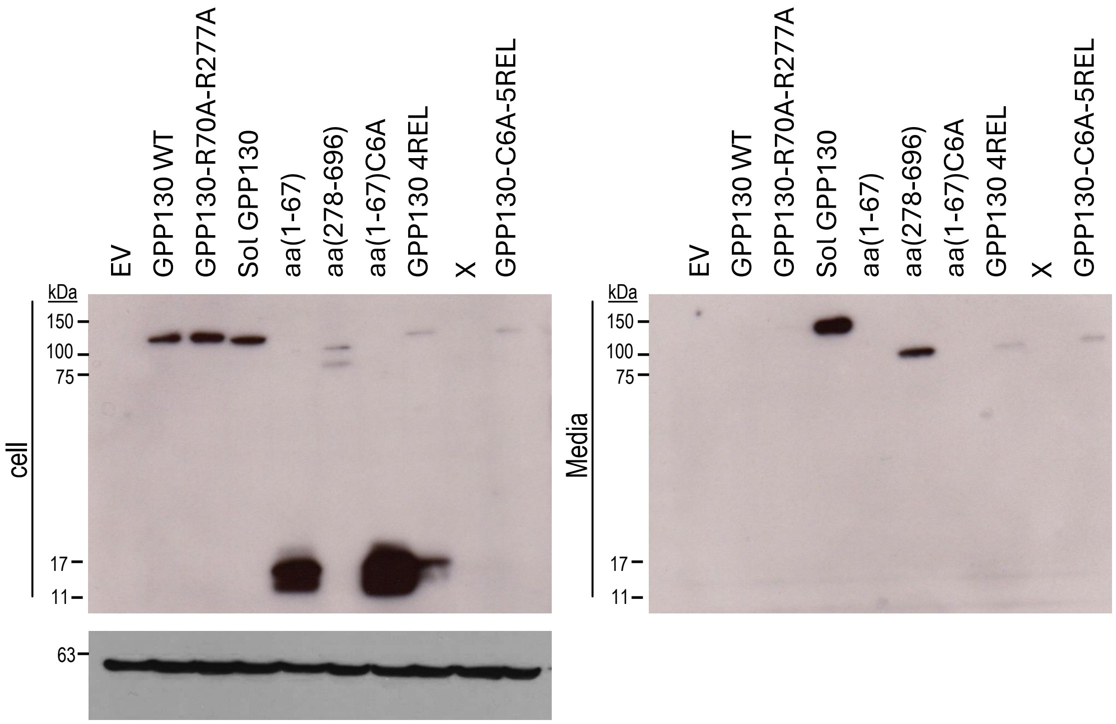

Supplemental Figure S1: Overexpression of all GPP130 constructs used in various proliferation experiment in A549 cells.

Western blot analysis of A549 cell lysate and media overexpressing different constructs of GPP130 tagged in the C terminal with a V5 tag, along with the empty vector (V5). “x” represents an empty well.

A

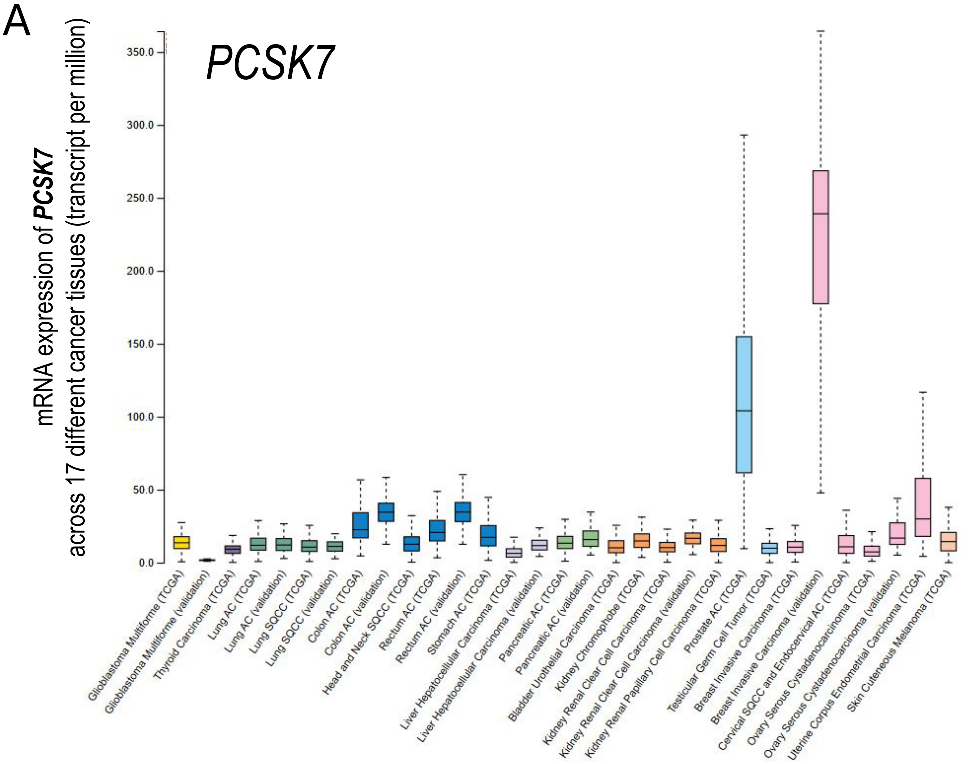
PCSK7
mRNA expression of PCSK7 across 17 different cancer tissues (transcript per million)
350.0
300.0
250.0
200.0
150.0
100.0
50.0
0.0
Glioblastoma Multiforme (TCGA)
Glioblastoma Multiforme (validation)
Thyroid Carcinoma (TCGA)
Lung AC (TCGA)
Lung AC (validation)
Lung SQCC (TCGA)
Lung SQCC (validation)
Colon AC (TCGA)
Colon AC (validation)
Head and Neck SQCC (TCGA)
Rectum AC (TCGA)
Rectum AC (validation)
Stomach AC (TCGA)
Liver Hepatocellular Carcinoma (TCGA)
Liver Hepatocellular Carcinoma (validation)
Pancreatic AC (TCGA)
Pancreatic AC (validation)
Bladder Urothelial Carcinoma (TCGA)
Kidney Chromophobe (TCGA)
Kidney Renal Clear Cell Carcinoma (TCGA)
Kidney Renal Clear Cell Carcinoma (validation)
Kidney Renal Papillary Cell Carcinoma (TCGA)
Prostate AC (TCGA)
Testicular Germ Cell Tumor (TCGA)
Breast Invasive Carcinoma (TCGA)
Breast Invasive Carcinoma (validation)
Cervical SQCC and Endocervical AC (TCGA)
Ovary Serous Cystadenocarcinoma (TCGA)
Ovary Serous Cystadenocarcinoma (validation)
Uterine Corpus Endometrial Carcinoma (TCGA)
Skin Cuteneous Melanoma (TCGA)

B

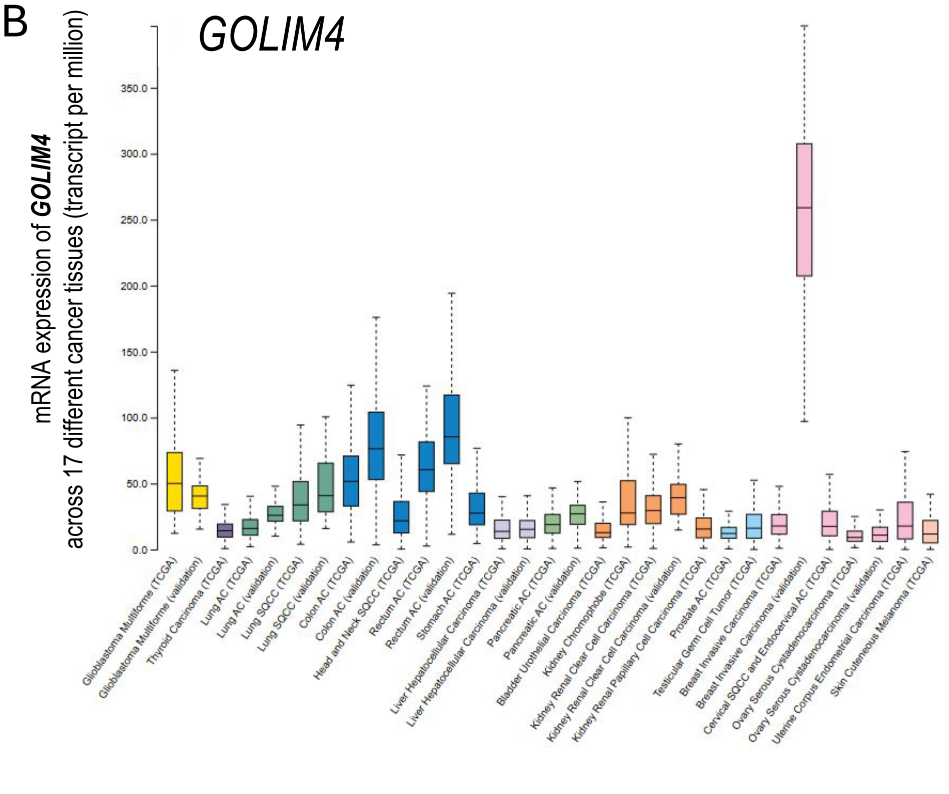
GOLIM4
mRNA expression of GOLIM4 across 17 different cancer tissues (transcript per million)
350.0
300.0
250.0
200.0
150.0
100.0
50.0
0.0
Glioblastoma Multiforme (TCGA)
Glioblastoma Multiforme (validation)
Thyroid Carcinoma (TCGA)
Lung AC (TCGA)
Lung AC (validation)
Lung SQCC (TCGA)
Lung SQCC (validation)
Colon AC (TCGA)
Colon AC (validation)
Head and Neck SQCC (TCGA)
Rectum AC (TCGA)
Rectum AC (validation)
Stomach AC (TCGA)
Liver Hepatocellular Carcinoma (TCGA)
Liver Hepatocellular Carcinoma (validation)
Pancreatic AC (TCGA)
Pancreatic AC (validation)
Bladder Urothelial Carcinoma (TCGA)
Kidney Chromophobe (TCGA)
Kidney Renal Clear Cell Carcinoma (TCGA)
Kidney Renal Clear Cell Carcinoma (validation)
Kidney Renal Papillary Cell Carcinoma (TCGA)
Prostate AC (TCGA)
Testicular Germ Cell Tumor (TCGA)
Breast Invasive Carcinoma (TCGA)
Breast Invasive Carcinoma (validation)
Cervical SQCC and Endocervical AC (TCGA)
Ovary Serous Cystadenocarcinoma (TCGA)
Ovary Serous Cystadenocarcinoma (validation)
Uterine Corpus Endometrial Carcinoma (TCGA)
Skin Cuteneous Melanoma (TCGA)

C

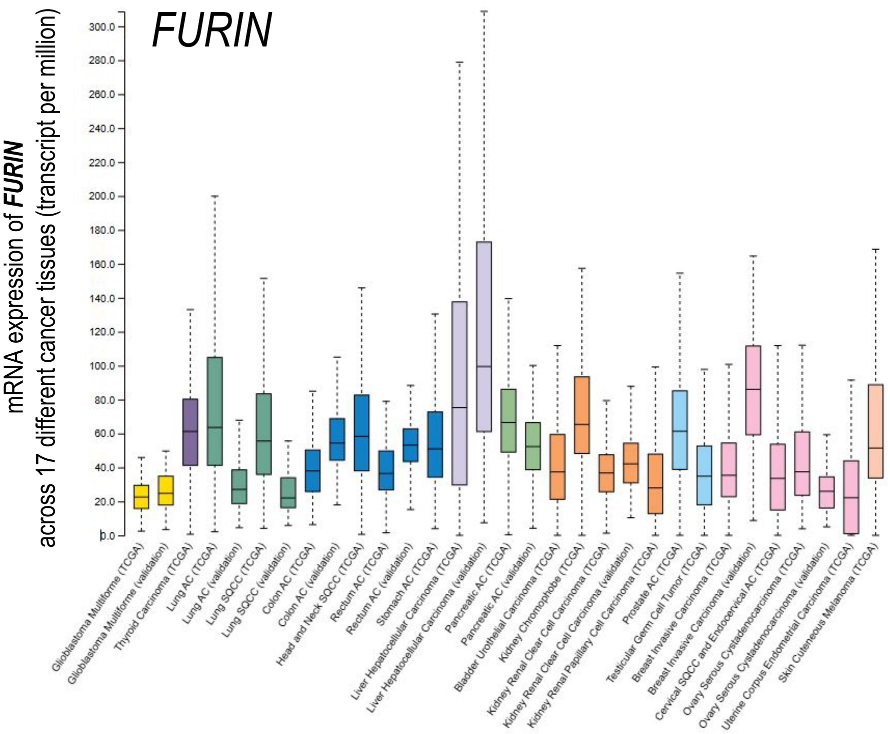

Supplemental Figure S2: mRNA expression levels across 17 human cancer tissues generated using the Cancer Genome Atlas (TGCA)

RNA expression overview of (A) *PCSK7* and (B) *GOLIM4 (gene name for* GPP130*) (C) FURIN* generated from the RNA seq data derived from the TGCA across different cancer tissues, depicting high expression of *PCSK7* and *GOLIM4* in invasive breast carcinoma.

Supplementary amino acid sequences of constructs used in the study:

GPP130
MGNGMCSRKQKR**IFQTLLLLTVVFGFLYGAMLYY**ELQTQLRKAEAVALKYQQHQESLSAQLQVVYEHRSR$_{70}$LEKSLQKERLEHKKAKEDFLVYKLEAQE
TLNKGRQDSNSRYSALNVQHQMLKSQHEELKKQHSDLEEEHRKQGEDFSRTFNDHKQKYLQLQQEKEQELSKLKETVYNLREENRQLRKAHQDIHTQLQ
DVKQQHKNLLSEHEQLVVTLEDHKSALAAAQTQVAEYKQLKDTLNRIPSLRKPDPAEQQNVTQVAHSPQGYNTAREKPTR$_{277}$EVQEVSRNNDVWQNHEAV
PGRAEDTKLYAPTHKEAEFQAPPEPIQQEVERREPEEHQVEEEHRKALEEEEMEQVGQAEHLEEEHDPSPEEQDREWKEQHEQREAANLLEGHARAEVYPS
AKPMIKFQSPYEEQLEQQRLAVQQVEEAQQLREHQEALHQQRLQGHLLRQQEQQQQQVAREMALQRQAELEEGRPQHQEQLRQQAHYDAMDNDIVQGA
EDQGIQGEEGAYERDNQHQDEAEGDPGNRHEPREQGPREADPESEADRAAVEDINPADDPNNQGEDEFEEAEQVREENLPDENEEQKQSNQKQENTEVEEH
LVMAGNPDQQEDNVDEQYQEEAEEEVQEDLTEEKKRELEHNAEETYGENDENTDDKNNDGEEQEVRDDNRPKGREEHYEEEEEEEEDGAAVAEKSHRRA
EMTGKPIPNPLLGLDST$_{696}$

GPP130-4REL
MGNGMCSRKQKR**IFQTLLLLTVVFGFLYGAMLYY**ELQTQLRKAEAVALKYQQHQESLSAQLQVVYERRRR$_{70}$ELKSLQKERLEHKKAKEDFLVYKLEAQE
TLNKGRQDSNSRYSALNVQHQMLKSQHEELKKQHSDLEEEHRKQGEDFSRTFNDHKQKYLQLQQEKEQELSKLKETVYNLREENRQLRKAHQDIHTQLQ
DVKQQHKNLLSEHEQLVVTLEDHKSALAAAQTQVAEYKQLKDTLNRIPSLRKPDPAEQQNVTQVAHSPQGYNTAREKPTREVQEVSRNNDVWQNHEAVPG
RAEDTKLYAPTHKEAEFQAPPEPIQQEVERREPEEHQVEEEHRKALEEEEMEQVGQAEHLEEEHDPSPEEQDREWKEQHEQREAANLLEGHARAEVYPSAK
PMIKFQSPYEEQLEQQRLAVQQVEEAQQLREHQEALHQQRLQGHLLRQQEQQQQQVAREMALQRQAELEEGRPQHQEQLRQQAHYDAMDNDIVQGAED
QGIQGEEGAYERDNQHQDEAEGDPGNRHEPREQGPREADPESEADRAAVEDINPADDPNNQGEDEFEEAEQVREENLPDENEEQKQSNQKQENTEVEEHLV
MAGNPDQQEDNVDEQYQEEAEEEVQEDLTEEKKRELEHNAEETYGENDENTDDKNNDGEEQEVRDDNRPKGREEHYEEEEEEEEDGAAVAEKSHRRAEM
TGKPIPNPLLGLDST$_{696}$

Sol-GPP130 (underlined sequence represents aa 1-34 of the N-terminal segment of PCSK9 including the signal peptide aa 1-30)
MGTVSSRRSWWPLPLLLLLLLLLGPAGARAQEDE$_{72}$KSLQKERLEHKKAKEDFLVYKLEAQETLNKGRQDSNSRYSALNVQHQMLKSQHEELKKQHSDLEE
EHRKQGEDFSRTFNDHKQKYLQLQQEKEQELSKLKETVYNLREENRQLRKAHQDIHTQLQDVKQQHKNLLSEHEQLVVTLEDHKSALAAAQTQVAEYKQ
LKDTLNRIPSLRKPDPAEQQNVTQVAHSPQGYNTAREKPTREVQEVSRNNDVWQNHEAVPGRAEDTKLYAPTHKEAEFQAPPEPIQQEVERREPEEHQVEEE
HRKALEEEEMEQVGQAEHLEEEHDPSPEEQDREWKEQHEQREAANLLEGHARAEVYPSAKPMIKFQSPYEEQLEQQRLAVQQVEEAQQLREHQEALHQQR
LQGHLLRQQEQQQQQVAREMALQRQAELEEGRPQHQEQLRQQAHYDAMDNDIVQGAEDQGIQGEEGAYERDNQHQDEAEGDPGNRHEPREQGPREADP
ESEADRAAVEDINPADDPNNQGEDEFEEAEQVREENLPDENEEQKQSNQKQENTEVEEHLVMAGNPDQQEDNVDEQYQEEAEEEVQEDLTEEKKRELEHN
AEETYGENDENTDDKNNDGEEQEVRDDNRPKGREEHYEEEEEEEEDGAAVAEKSHRRAEMTGKPIPNPLLGLDST$_{659}$

GPP130 aa(278-696) (underlined sequence represents aa 1-33 of the N-terminal segment of PCSK9 including the signal peptide aa 1-30)
MGTVSSRRSWWPLPLLLLLLLLLGPAGARAQEDE$_{278}$VQEVSRNNDVWQNHEAVPGRAEDTKLYAPTHKEAEFQAPPEPIQQEVERREPEEHQVEEEHRKAL
EEEEMEQVGQAEHLEEEHDPSPEEQDREWKEQHEQREAANLLEGHARAEVYPSAKPMIKFQSPYEEQLEQQRLAVQQVEEAQQLREHQEALHQQRLQGHL
LRQQEQQQQQVAREMALQRQAELEEGRPQHQEQLRQQAHYDAMDNDIVQGAEDQGIQGEEGAYERDNQHQDEAEGDPGNRHEPREQGPREADPESEAD
RAAVEDINPADDPNNQGEDEFEEAEQVREENLPDENEEQKQSNQKQENTEVEEHLVMAGNPDQQEDNVDEQYQEEAEEEVQEDLTEEKKRELEHNAEETY
GENDENTDDKNNDGEEQEVRDDNRPKGREEHYEEEEEEEEDGAAVAEKSHRRAEMTGKPIPNPLLGLDST$_{696}$

aa(1-67)
MGNGMC$_{6}$SRKQKR**IFQTLLLLTVVFGFLYGAMLYY**ELQTQLRKAEAVALKYQQHQESLSAQLQVVYER$_{67}$

aa(1-67) C6A
MGNGMA$_{6}$SRKQKR**IFQTLLLLTVVFGFLYGAMLYY**ELQTQLRKAEAVALKYQQHQESLSAQLQVVYER$_{67}$

GPP130-5REL-C6A
MGNGMA$_{6}$SRKQKR**IFQTLLLLTVVFGFLYGAMLYY**ELQTQLRKAEAVALKYQQHQESLSAQLQVVYEHRSRLEKSLQKERLEHKKAKEDFLVYKLEAQE
TLNKGRQDSNSRYSALNVQHQMLKSQHEELKKQHSDLEEEHRKQGEDFSRTFNDHKQKYLQLQQEKEQELSKLKETVYNLREENRQLRKAHQDIHTQLQ
DVKQQHKNLLSEHEQLVVTLEDHKSALAAAQTQVAEYKQLKDTLNRIPSLRKPDPAEQQNVTQVAHSPQGYNTARRRRRR$_{277}$ELQEVSRNNDVWQNHEAV
PGRAEDTKLYAPTHKEAEFQAPPEPIQQEVERREPEEHQVEEEHRKALEEEEMEQVGQAEHLEEEHDPSPEEQDREWKEQHEQREAANLLEGHARAEVYPS
AKPMIKFQSPYEEQLEQQRLAVQQVEEAQQLREHQEALHQQRLQGHLLRQQEQQQQQVAREMALQRQAELEEGRPQHQEQLRQQAHYDAMDNDIVQGA
EDQGIQGEEGAYERDNQHQDEAEGDPGNRHEPREQGPREADPESEADRAAVEDINPADDPNNQGEDEFEEAEQVREENLPDENEEQKQSNQKQENTEVEEH
LVMAGNPDQQEDNVDEQYQEEAEEEVQEDLTEEKKRELEHNAEETYGENDENTDDKNNDGEEQEVRDDNRPKGREEHYEEEEEEEEDGAAVAEKSHRRA
EMTGKPIPNPLLGLDST

GPP130-R70A-R277A
MGNGMCSRKQKR**IFQTLLLLTVVFGFLYGAMLYY**ELQTQLRKAEAVALKYQQHQESLSAQLQVVYEHRSA$_{70}$LEKSLQKERLEHKKAKEDFLVYKLEAQE
TLNKGRQDSNSRYSALNVQHQMLKSQHEELKKQHSDLEEEHRKQGEDFSRTFNDHKQKYLQLQQEKEQELSKLKETVYNLREENRQLRKAHQDIHTQLQ
DVKQQHKNLLSEHEQLVVTLEDHKSALAAAQTQVAEYKQLKDTLNRIPSLRKPDPAEQQNVTQVAHSPQGYNTAREKPTA$_{277}$EVQEVSRNNDVWQNHEAV
PGRAEDTKLYAPTHKEAEFQAPPEPIQQEVERREPEEHQVEEEHRKALEEEEMEQVGQAEHLEEEHDPSPEEQDREWKEQHEQREAANLLEGHARAEVYPS
AKPMIKFQSPYEEQLEQQRLAVQQVEEAQQLREHQEALHQQRLQGHLLRQQEQQQQQVAREMALQRQAELEEGRPQHQEQLRQQAHYDAMDNDIVQGA
EDQGIQGEEGAYERDNQHQDEAEGDPGNRHEPREQGPREADPESEADRAAVEDINPADDPNNQGEDEFEEAEQVREENLPDENEEQKQSNQKQENTEVEEH
LVMAGNPDQQEDNVDEQYQEEAEEEVQEDLTEEKKRELEHNAEETYGENDENTDDKNNDGEEQEVRDDNRPKGREEHYEEEEEEEEDGAAVAEKSHRRA
EMTGKPIPNPLLGLDST

Underlined bold sequences represent the transmembrane domain of GPP130.
